# A case report of atypical teratoid/rhabdoid tumor and pituitary adenoma collision tumor in the sellar region

**DOI:** 10.3389/fsurg.2025.1612035

**Published:** 2025-11-26

**Authors:** Qing Li, Li Wang, Yang Chen, Rui Luo, Jian-shan Wu, Ying Li, Ping-ping Li

**Affiliations:** 1 Kunming University of Science and Technology School of Medicine, Kunming, China; 2 Department of Pathology, The First People's Hospital of Yunnan Province / The Affiliated Hospital of Kunming University of Science and Technology, Kunming, China

**Keywords:** central nervous system tumors, atypical teratoid/rhabdoid tumor (AT/RT), pituitary adenoma (PA), collision tumor, INI-1

## Abstract

Collision tumor in the sellar region is extremely rare, and atypical teratoid/rhabdoid tumor (AT/RT) in the sellar region combined with pituitary adenoma has not been reported in the literature. AT/RT is a rare embryonal tumor of the central nervous system with extremely high malignancy and diverse histological morphology. The tumor cells can differentiate into primitive neuroectodermal, mesenchymal, or epithelial lineages and are characterized by rhabdoid cells. AT/RT mostly occurs in children, and its incidence in adults is extremely low. Here, we report a case of a collision tumor in the sellar region consisting of an atypical teratoid/rhabdoid tumor and a pituitary adenoma. The patient was a 33-year-old female who first presented with a headache for seven days and decreased vision in the left eye for four days. The initial imaging diagnosis was invasive pituitary macroadenoma, and partial resection of the pituitary lesion was performed under neuroendoscopy through the nasal cavity and sphenoid sinus. Postoperative pathology revealed the coexistence of an atypical teratoid/rhabdoid tumor and a pituitary adenoma. The diagnosis of AT/RT is challenging due to the patient's very uncommon age, non-specific imaging findings, unusual histological pattern, and complex tissue composition.

## Introduction

Two or more histopathologically distinct tumors coexisting in the same anatomical region are referred to as collision tumors. Collision tumors are extremely rare in the sellar region, with only a few published case reports available. Most reported cases involve pituitary adenoma (PA) coexisting with Rathke's cleft cyst (RCC) or craniopharyngioma (CP). Cases of PA coexisting with gangliocytoma (GC), meningioma, schwannoma, chondroma, or plasmacytoma have been rarely reported. To date, no reports have documented PA coexisting with an atypical teratoid/rhabdoid tumor (AT/RT), and the literature contains only one instance of a collision tumor involving CP and AT/RT (1).

According to epidemiological surveys conducted in Europe and the US, pituitary adenomas are the most prevalent tumors in the sellar region, accounting for approximately 84.6% of sellar tumors and 10%–15% of intracranial tumors, with an incidence of 1–8 per 100,000 (2). Pituitary adenomas originate in the anterior pituitary gland, and the vast majority are benign (1). However, a small subset of pituitary adenomas can invade critical structures such as the cavernous sinus, internal carotid artery, and optic chiasm. In rare cases, they may also exhibit metastatic potential, making complete surgical resection challenging. Even after complete removal, some pituitary adenomas have a risk of recurrence. Clinically, pituitary adenomas are classified as functional pituitary adenomas (FPAs), which present with characteristic symptoms (such as acromegaly or Cushing's disease), and non-functional pituitary adenomas (NFPAs), which are often associated with gonadotropin-secreting cells. Radiologically, they are categorized as microadenomas (<10 mm) or macroadenomas (≥10 mm). Recently, the term “adenoma” has been replaced by “pituitary neuroendocrine tumors (PitNETs)” to better reflect their biological behavior.

Malignant tumors of the central nervous system (CNS) known as AT/RTs are exceedingly rare and highly aggressive (3). AT/RT was first identified in 1996 (4) and was classified by the World Health Organization in 2016 as a subclass of embryonal CNS tumors, designated as grade 4 tumors (5). Characteristic histomorphological features of this tumor include cell clusters with rhabdomyosiform characteristics, such as prominent eosinophilic nucleoli, abundant cytoplasm with distinct globular eosinophilic cytoplasmic inclusions (6), and well-defined cell borders (5). AT/RT primarily occurs in children and is extremely rare in adults (7). With an average annual incidence of 0.07 per 100,000, AT/RT accounts for 1.6% of all pediatric CNS tumors and 4.4% of CNS tumors in children aged 0–5 years, according to the US Central Brain Tumor Registry (8). Fewer than 50 cases of adult AT/RT have been documented in the literature, making it an exceptionally rare malignancy (9). The sellar region is the most frequent site of adult AT/RT occurrence (9).

## Case report

**Figure 1:**
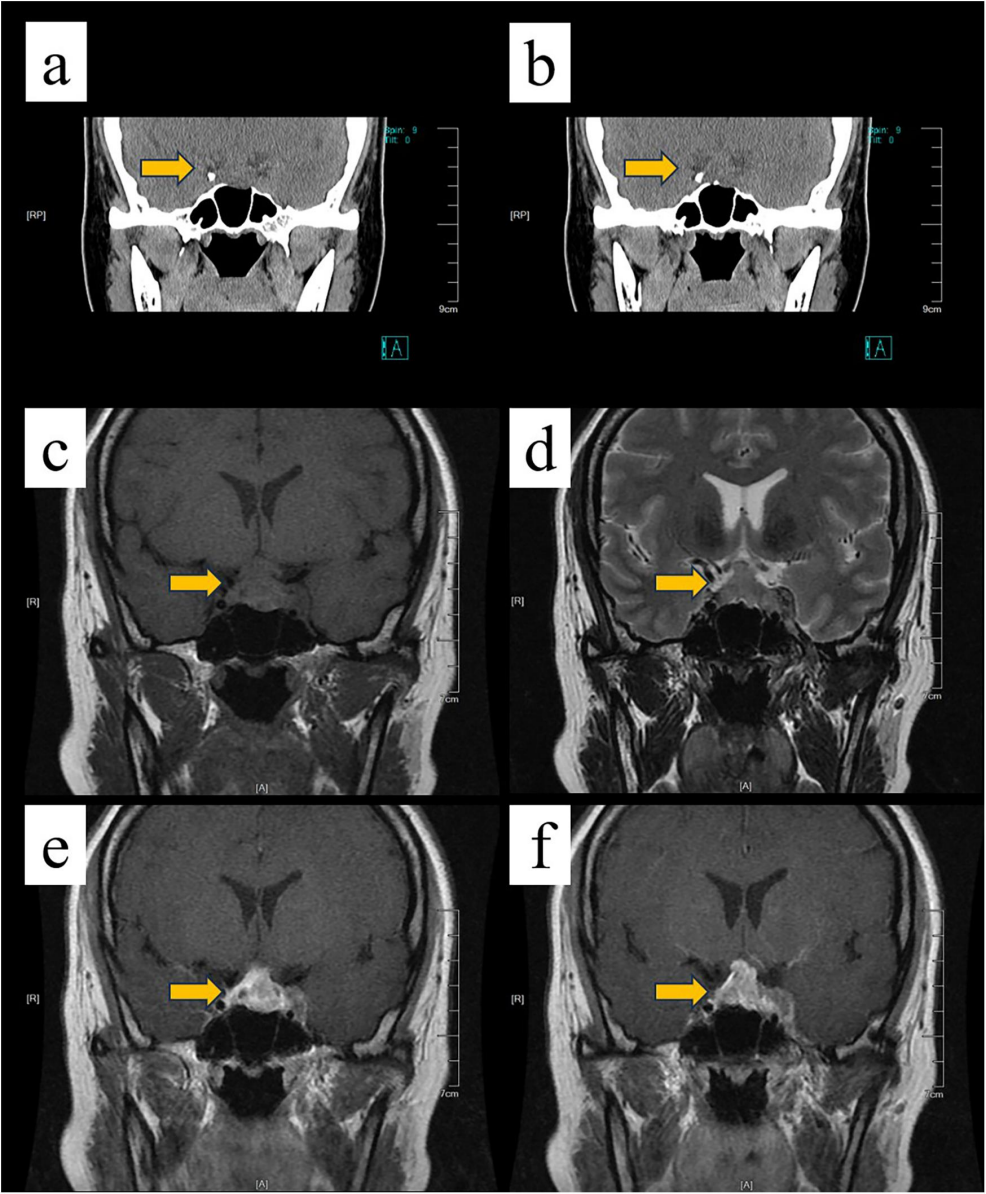
Preoperative neuroimaging findings of the sellar region mass. Cranial computed tomography (CT) scan **(a,b)** shows a thick soft tissue mass in the sella turcica. Note the absence of calcification within the mass, as well as the compression and resorption of the surrounding bone (arrow). Magnetic resonance imaging (MRI) sequences **(c,d)**. T1-weighted image **(c)**: The right and middle portions of the mass are isointense, while the left portion is slightly hyperintense. T2-weighted image **(d)**: The right and middle portions show an isointense to slightly hyperintense signal, and the left portion is isointense. Contrast-enhanced **(e,f)**: The lesion enhances significantly. The enhancement is heterogeneous in the right and middle portions and mild in the left portion. The mass envelops the pituitary gland and stalk, and the left internal carotid artery appears embedded (arrowhead).

A 33-year-old woman was admitted to the hospital with a headache for seven days, accompanied by decreased vision in the left eye for four days, and no personal or family history of tumors. On admission, the patient was conscious, with limited abduction of the left eye and a temporal visual field defect in the left eye. Bilateral pupils were equal and round with a diameter of 2.5 mm, sensitive to light reflex, with no obvious tongue or facial paralysis, no limitation of limb movement, and limb muscle strength at grade IV. The patient's hormonal laboratory findings on admission were as follows (reference ranges in parentheses): Estradiol 37 pmol/L (Follicular phase: 77.07–921.17; Mid-cycle: 139.46–2,381.83; Luteal phase: 77.07–1,145.04; Postmenopausal: <36.7), LH 0.42 IU/L (Early follicular phase: 1.8–11.78; Ovulatory phase: 7.59–89.08; Luteal phase: 0.56–14.00; Postmenopausal: 5.16–61.99), FSH 0.91 IU/L (Follicular phase: 3.03–8.08; Ovulatory phase: 2.55–16.69; Luteal phase: 1.38–5.47; Postmenopausal: 26.72–133.41), PRL 532.40 mIU/L (108.78–557.00), Progesterone <0.3 nmol/L (Follicular phase: 0.318–0.954; Luteal phase: 3.816–50.562; Postmenopausal: <0.318), Testosterone <0.45 nmol/L (0.38–1.97); TSH 0.051 mIU/L (0.3–4.2), T4 62.4 nmol/L (55–138), T3 1.84 nmol/L (1.3–2.5), FT4 7.8 pmol/L (11.5–22.7), FT3 3.56 pmol/L (3.5–6.5). The circadian cortisol and ACTH levels were: 21.74 µg/dl and 3.91 pg/ml at 12:00 AM; 15.03 µg/dl and 3.07 pg/ml at 8:00 AM (ACTH reference: 7.2–63.3 pg/ml); 0.685 µg/dl and 2.07 pg/ml at 4:00 PM. GH was 0.183 ng/ml (<3). These results indicated preoperative deficiencies in thyrotropin, thyroid hormone, and serum free thyroxine, as well as a low morning serum corticotropin level. Preoperative cranial CT and MRI revealed a large, heterogeneously enhancing mass in the sellar region (Figure 1). The lesion exhibited aggressive features, including bony erosion of the sella turcica and encasement of the left internal carotid artery. These imaging characteristics were highly suggestive of an invasive neoplasm, warranting surgical intervention. Based on these findings, an invasive pituitary macroadenoma was considered. After full surgical preparation, neuroendoscopic pituitary lesion resection was performed via the transnasal-sphenoidal route under general anesthesia. During the operation, the sellar floor bone was found to be eroded, appearing thin, and the tumor was located in the sellar and suprasellar regions. The tumor measured approximately 1.5 cm × 2 cm × 2 cm and lacked an obvious capsule. It was firm in consistency, resembling rotten fish meat, with a rich blood supply. The tumor had invaded and penetrated the left cavernous sinus. Due to the firm texture of the tumor within the left cavernous sinus, complete removal was not feasible, and partial lesion resection was performed.

**Figure 2:**
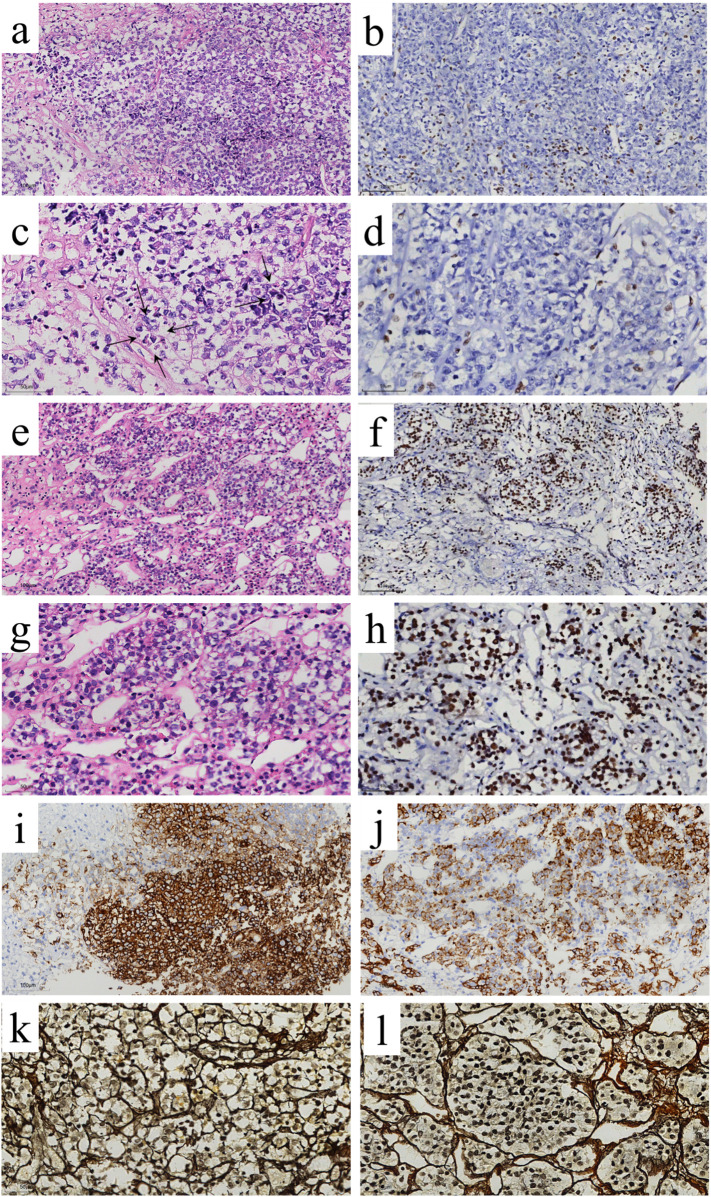
Histopathological and immunohistochemical features of the AT/RT and pituitary adenoma (PA) collision tumor. Histology (H&E staining): The AT/RT component **(a,c)** is composed of hyperchromatic small cells with a high nuclear-to-cytoplasmic ratio, interspersed with rhabdoid cells (arrows). In contrast, the PA component **(e,g)** consists of eosinophilic small round cells arranged in nests and organoids. Immunohistochemistry (INI1): Tumor cells in the AT/RT component show a complete loss of nuclear INI1 expression **(b,d)**, while nuclear INI1 staining is retained in the PA component **(f,h)**. Ancillary Stains: EMA immunohistochemistry shows focal positivity in the AT/RT component **(i)**. LCK is positive in the PA component **(j)**. Reticular fiber staining highlights a diffuse growth pattern in AT/RT **(k)** vs. a nested architecture in PA **(l)**. Original magnifications: ×200 **(a,b,e,f,i,j)**; ×400 **(c,d,g,h,k,l)**.

Histologic examination revealed that the tumor had two distinct components (Figure 2). The PA component was arranged in nests and organoid structures, composed of small, rounded cells with mild morphological features, eosinophilic cytoplasm, small nuclei, and an inconspicuous nucleolus. The AT/RT component exhibited diffuse tumor cell proliferation against a background of extensive necrosis. The tumor cells were markedly larger than those in the PA component and displayed significant cellular atypia. They had basophilic and scant cytoplasm, thickened nuclear membranes, and a relatively high nuclear-to-cytoplasm ratio. The tumor cell nuclei were irregular and vesicular, with a prominent acidophilic nucleolus. In some tumor cells, the eosinophilic cytoplasm appeared to displace the nucleus to one side.

The tumor immunohistochemistry showed different expression patterns in the two components. The PA component was positive for pan-keratin, LCK, chromogranin A (CgA), synaptophysin (Syn), CD56, glial fibrillary acidic protein (GFAP), PIT-1, INI-1, BRG-1, GH, and PRL, with focal positivity for epithelial membrane antigen (EMA). TSH, ACTH, FSH, luteinizing hormone (LH), T-PIT, and SF-1 were negative. The Ki-67 labeling index was approximately 3%, and P53 exhibited a wild-type expression pattern. The AT/RT component was positive for BRG-1 and partially positive for EMA and Glypican-3. INI-1 staining, although positive in adjacent inflammatory cells, was completely negative in the tumor cells. Immunostaining for GFAP, pan-keratin, LCK, CgA, Syn, CD56, ACTH, PRL, TSH, FSH, GH, LH, PIT-1, T-PIT, and SF-1 was entirely negative. P53 was expressed in a mutant form, and the Ki-67 labeling index was approximately 80%. In both tumor components, CD30, leukocyte common antigen (LCA), ALK, vimentin (VIM), S-100 protein, desmin, CD117, myogenin, MyoD1, OCT3/4, PLAP, and human chorionic gonadotropin (HCG) were all negative (Figure 2).

Based on the HE morphology and immunohistochemistry results, the pathological diagnosis was AT/RT coexisting with prolactin-growth hormone-type pituitary neuroendocrine tumor (Pit-NET)/PA.

Subsequently, under the microscope, the tumor components of AT/RT and PA were circled with different color markers on the HE section. The paraffin-embedded tissue sections of the patient's tumor were then cut, and the tissue sections corresponding to AT/RT and PA were extracted and placed into separate test tubes for transcriptome sequencing. Notably, despite adequate sequencing coverage and quality, no definitive pathogenic point mutations or small insertions/deletions were identified within the coding region of the SMARCB1/INI1 gene. Given that INI-1 protein loss remains a cornerstone for diagnosis, we further explored the sequencing data to investigate other molecular features.

**Figure 3:**
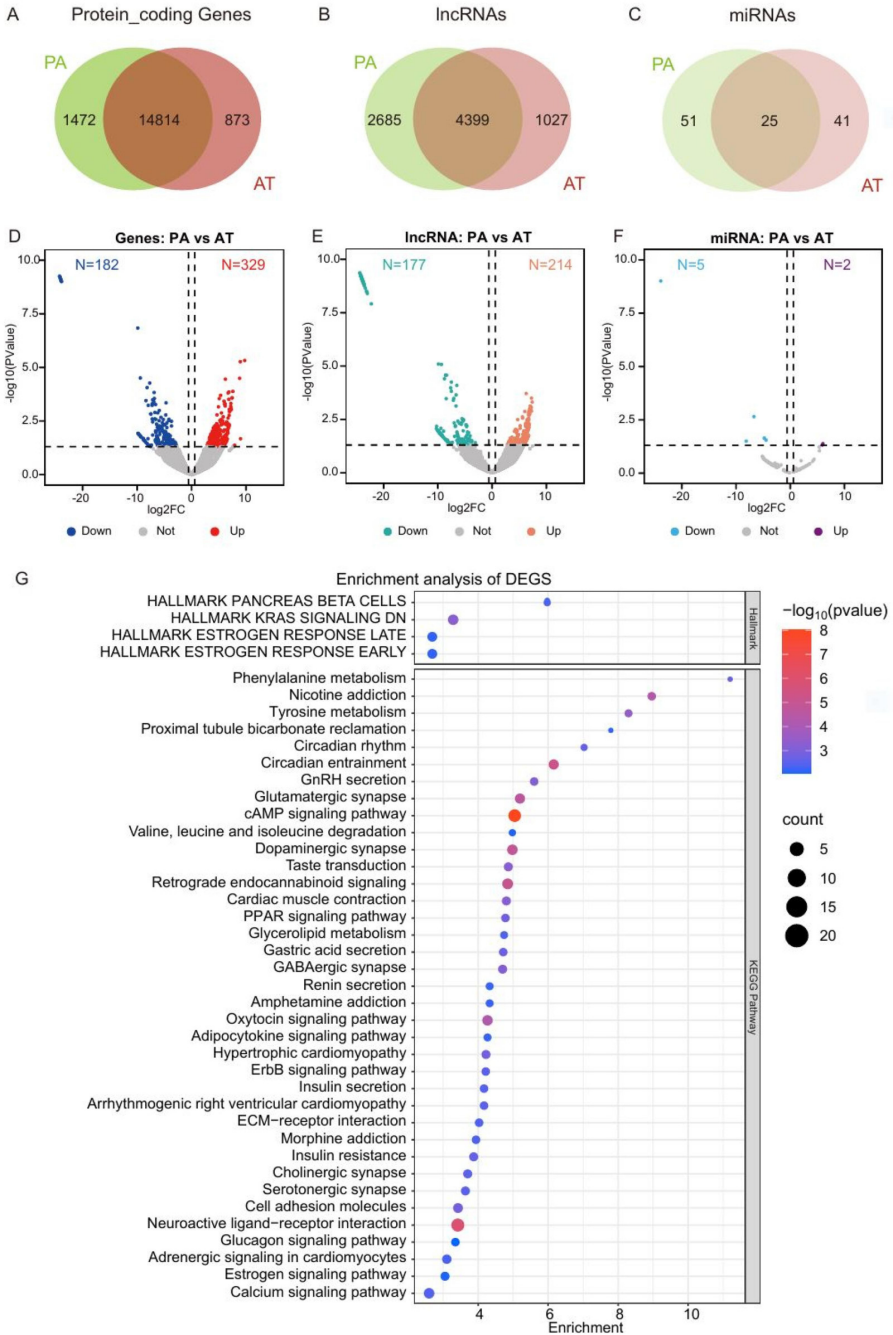
Expression of protein-coding genes, long non-coding RNAs (lncRNAs), and microRNAs (miRNAs) in pituitary adenoma (PA) and atypical teratoid/rhabdoid tumor (AT/RT) **(A–C)**. Differential expression analysis of genes **(D)**, lncRNAs **(E)**, and miRNAs **(F)**. Functional enrichment analysis of differentially expressed genes **(G)**.

Bioinformatic analysis revealed 14,814 common protein-coding genes, 4,399 common lncRNAs, and 25 common miRNAs shared between PA and AT/RT. PA had 1,472 specific protein-coding genes, 2,685 specific lncRNAs, and 51 specific miRNAs, whereas AT/RT had 873 specific protein-coding genes, 1,027 specific lncRNAs, and 41 specific miRNAs. Gene differential analysis identified 182 downregulated protein-coding genes, 177 downregulated lncRNAs, and 5 downregulated miRNAs, along with 329 upregulated protein-coding genes, 214 upregulated lncRNAs, and 2 upregulated miRNAs. According to the enrichment analysis of differentially expressed genes (DEGs) in the Hallmark gene set (Figure 3), AT/RT was primarily enriched in pancreatic beta cells, KRAS signaling downregulation, late estrogen response, early estrogen response signaling pathways, phenylalanine metabolism, the cAMP signaling pathway, and the calcium signaling pathway.

The patient's postoperative course was complicated by a slight reduction in headache, limited left eye abduction, a persistent left temporal visual field defect, bilateral anisocoria, and new-onset diabetes insipidus. Hormonal evaluation on the fourth postoperative day revealed panhypopituitarism, with most values falling below the reference ranges: estradiol <37 pmol/L (follicular phase: 77.07–921.17), LH 0.05 IU/L (1.8–11.78), FSH 0.25 IU/L (3.03–8.08), PRL 13.14 mIU/L (108.78–557.00), progesterone <0.3 nmol/L (0.318–0.954), testosterone <0.45 nmol/L (0.38–1.97); TSH 0.013 mIU/L (0.3–4.2), T4 59.9 nmol/L (55–138), T3 0.82 nmol/L (1.3–2.5), FT4 9.8 pmol/L (11.5–22.7), FT3 2.01 pmol/L (3.5–6.5); 8:00 AM cortisol was critically low at 0.475 µg/dl (5–25) with ACTH at 1.09 pg/ml (7.2–63.3). A disrupted circadian rhythm was noted with cortisol at 24.47 µg/dl at 16:00 and 2.61 µg/dl at 24:00. GH was <0.030 ng/ml (<3). Marked decreases were observed in GH, TSH, thyroid hormones, PRL, and morning serum cortisol and ACTH levels compared to preoperative states.

### Diagnosis and treatment

The patient underwent preoperative hormone replacement therapy along with symptomatic and supportive care, and completed all relevant examinations. Surgery was performed three days after admission. Following pathological confirmation of a malignant tumor, the patient received intravenous ondansetron and oral temozolomide chemotherapy. Ondansetron was administered at a dose of 8 mg once daily at 8:00 AM, while temozolomide was given at 300 mg once daily at 8:00 AM. The patient was discharged 25 days postoperatively in stable condition.

### Medication guidance

At discharge, the patient was prescribed oral levothyroxine sodium at 25 µg once daily at 8:00 AM, and prednisone at 2.5 mg once daily in the morning (8:00 AM) and 5 mg once daily in the afternoon (4:00 PM). She was instructed to undergo laboratory tests at a local hospital every 1–2 weeks, including assessments of sex hormones, thyroid hormones, 8:00 AM serum cortisol, and 8:00 AM ACTH. Dosage adjustments were to be guided by test results, and the patient was advised not to self-adjust or discontinue any medication without medical consultation.

### Follow-up plan

The patient was scheduled to continue radiotherapy and chemotherapy after discharge. A follow-up contrast-enhanced MRI of the head was recommended after three cycles of chemotherapy.

### Follow-up

After discharge, the patient received radiotherapy at a local hospital, with a total dose of 54–59 Gy, 1.8–2.0 Gy per fraction, for approximately 30 fractions, combined with oral temozolomide chemotherapy. The patient did not undergo relevant tests, so the treatment effect could not be determined. During the treatment, the patient's condition was unsatisfactory, and she died five months after the operation.

## Discussion

Atypical teratoid/rhabdoid tumor (AT/RT) is a highly malignant embryonal neoplasm predominantly seen in infants. Its occurrence in the sellar region of an adult is exceedingly rare, and its presentation as a collision tumor with a pituitary adenoma (PA) is, to our knowledge, unprecedented. The clinical and radiological features of sellar AT/RTs are non-specific and frequently overlap with those of invasive pituitary adenomas, often posing diagnostic challenges preoperatively (9–12). In the present case, the rapidly progressive symptomatology over one week highlighted the aggressive behavior of the tumor.

The diagnosis of AT/RT relies on an integrated pathological approach. Although histomorphology may reveal characteristic rhabdoid cells, the tumor frequently consists mainly of small round cells, as observed in our case (13, 14). Critically, the loss of nuclear INI1 protein expression on immunohistochemistry (IHC) represents a genetic hallmark and remains the diagnostic gold standard per the WHO classification (5, 15, 16). In this study, we identified a discordance between the loss of INI1 protein expression by IHC and the absence of detectable mutations in the coding region of the SMARCB1 gene. This phenomenon, documented in a minority of AT/RT cases, may be explained by several mechanisms: 1) large genomic deletions or complex rearrangements not reliably captured by conventional targeted sequencing; 2) epigenetic silencing, such as promoter hypermethylation, requiring dedicated methylation assays for confirmation; and 3) tumor heterogeneity, where the tissue sample used for DNA extraction may have contained INI1-negative but SMARCB1 wild-type tumor cells (17, 18). Notwithstanding this molecular discrepancy, the definitive loss of INI1 by IHC confirms the diagnosis.

Beyond diagnostic confirmation, our bioinformatic analysis yielded substantial additional value. It served, first, as a tool for exclusionary diagnosis, by ruling out molecular hallmarks of other histologically similar malignancies—such as IDH1/2 mutations in high-grade gliomas and H3F3A mutations in certain embryonal tumors—thereby consolidating the diagnosis of AT/RT. Second, it enabled the discovery of novel insights: we identified mutations in genes including KRAS, which may cooperatively contribute to tumor pathogenesis. Additionally, evidence of enrichment in the cAMP and calcium signaling pathways in the PA component provides a potential clue to the origin of this collision tumor. These observations suggest that INI-1 loss in the present AT/RT may result from an unconventional non-coding mutation or epigenetic event—a hypothesis meriting further investigation.

The management of this complex case was underpinned by a multidisciplinary team (MDT) involving neurosurgeons, pathologists, and oncologists. The establishment of a precise pathological diagnosis—integrating morphological, immunohistochemical, and molecular data—was foundational to all subsequent therapeutic decisions. It informed the neurosurgeons' aim for maximal safe resection and guided the oncologists in devising an adjuvant regimen that included temozolomide-based chemotherapy. For aggressive neoplasms such as AT/RT, a multimodal strategy encompassing maximal resection, chemotherapy, and radiotherapy remains essential to improving survival (19–21).

In summary, we report a uniquely rare case of a sellar collision tumor composed of PA and AT/RT. Our report underscores the critical role of INI1 IHC in diagnosing AT/RT, particularly in the setting of inconclusive genetic sequencing. Although the bioinformatic analysis did not elucidate the mechanism of SMARCB1 inactivation, it provided valuable exclusionary information and generated new hypotheses concerning the molecular drivers of this tumor. This case further emphasizes the necessity of an MDT framework and a comprehensive pathological workup in the diagnosis and management of rare intracranial neoplasms.

## Conclusion

Collision tumor of AT/RT with PA is extremely rare in the sellar region. Its definite diagnosis mainly depends on histopathological and immunohistochemical staining, and it needs to be differentiated from other common collision tumors of PA. Imaging examination can be helpful. To the best of our knowledge, this is the first reported case of a collision tumor with AT/RT in the sellar region combined with PA in the literature, providing new data. In sellar malignant tumors in adults, the differential diagnosis of AT/RT should be considered. Due to the small number of cases, multi-center and comprehensive studies are needed to clarify the mechanism of occurrence and development of collision tumor of AT/RT with PA, so as to develop a more complete diagnostic basis and a better treatment plan for prognosis.

## Data Availability

The datasets presented in this article are not readily available because of ethical and privacy restrictions. Requests to access the datasets should be directed to the corresponding author.
